# Cross-Scanner Harmonization of AI/DL Accelerated Quantitative Bi-Parametric Prostate MRI †

**DOI:** 10.3390/s25185858

**Published:** 2025-09-19

**Authors:** Dariya Malyarenko, Scott D. Swanson, Jacob Richardson, Suzan Lowe, James O’Connor, Yun Jiang, Reve Chahine, Shane A. Wells, Thomas L. Chenevert

**Affiliations:** 1 Department of Radiology, University of Michigan Medical School, Ann Arbor, MI 48109, USA; 2 Department of Urology, University of Michigan Medical School, Ann Arbor, MI 48109, USA

**Keywords:** bi-parametric MRI, apparent diffusion coefficient (ADC), T_2_, relaxation, quantitative parameter mapping, AI/DL-accelerated image reconstruction, multi-vendor harmonization, prostate imaging reporting data systems (PIRADS)

## Abstract

Clinical application of AI/DL-aided acquisitions for quantitative bi-parametric (q-bp)MRI requires validation and harmonization across vendor platforms. An AI/DL-accelerated q-bpMRI, including 5-echo T_2_ and 4-b-value apparent diffusion coefficient (ADC) mapping, was implemented on two 3T clinical scanners by two vendors alongside the qualitative standard-of-care (SOC) MRI protocols for six patients with biopsy-confirmed prostate cancer (PCa). AI/DL versus SOC bpMRI image quality was compared for MR-visible PCa lesions on a 4-point Likert-like scale. Quantitative validation and protocol bias assessment were performed using a multiparametric phantom with reference T_2_ and diffusion kurtosis values mimicking prostate tissue ranges. Six-minute q-bpMRI achieved acceptable diagnostic quality comparable to the SOC. Better SNR was observed for DL/AI versus SOC ADC with method-dependent distortion susceptibility and resolution enhancement. The measured biases were unaffected by AI/DL reconstruction and related to acquisition protocol parameters: constant for spin-echo T_2_ (−7 ms to +5 ms) and ADC (4b-fit: −0.37 µm^2^/ms and 2b-fit: −0.19 µm^2^/ms), while nonlinear for echo-planar T_2_ (−37 ms to +14 ms). Measured phantom ADC bias dependence on b-value range was consistent with that observed for PCa lesions. Bias correction harmonized lesion T_2_ and ADC values across different AI/DL-aided q-bpMRI acquisitions. The developed workflow enables harmonization of AI/DL-accelerated quantitative T_2_ and ADC mapping in multi-vendor clinical settings.

## 1. Introduction

Current clinical management of patients with prostate cancer (PCa) relies on costly and painful multi-core needle biopsies performed for both diagnosis and active surveillance. Due to the relatively low occurrence of high-risk cancers, 80–90% PCa patients could potentially be spared unnecessary biopsies [1,2]. The standard-of-care (SOC) multiparametric (mp)MRI improves quality of life, [3], and qualitative interpretation according to Prostate Imaging Reporting and Data System (PIRADS) [4] provides excellent sensitivity for PCa detection [5,6]. However, modest specificity of subjective PIRADS scores necessitates biopsy confirmation for PRADS > 2 lesions, including a large portion of indolent cancers. For lesion assessment in the peripheral zone where the majority of PCa occur [2,7], bi-parametric (bp)MRI is typically sufficient. In addition to the T2-weighted anatomical scan, the diffusion weighted imaging (DWI) component of the bpMRI SOC exam includes calculation of an apparent diffusion coefficient (ADC) and qualitative examination of high b-value (>1400 s/mm^2^) images for signs of impeded diffusion. ADC maps are routinely generated on the scanner with different hospitals using variable b-ranges that affect derived values [5,8], complicating quantitative ADC interpretation.

Imaging studies that add assessment of quantitative diffusion parameter [8,9,10] and T_2_ tissue relaxation [11,12,13] improve specificity for clinically significant (cs)PCa [8,9,14] that tends to have lower ADC and T_2_ values, which may reduce the need for biopsies. However, comprehensive quantitative bpMRI studies typically require lengthy (multi-b and multi-TE) custom acquisitions [12,13,14,15] that limit their practical utility for clinical SOC examinations. PCa MRI protocol optimization is subject to a balance between acquisition time, spatial resolution, and signal-to-noise ratio (SNR). Hence, diffusion and relaxation metric values derived from abbreviated acquisition protocols are inevitably biased, depending on the applied models [8,10,11,12,15,16,17] and acquisition settings (e.g., b-value or TE ranges) [14,18,19]. These biases would also vary across MRI vendor platforms used in multi-site clinical trials and for longitudinal follow-up of patients on active surveillance in clinical settings [5,8,14]. Thus, measured ADC and T_2_ values require harmonization for quantitative comparison across studies and vendor platforms to establish uniform thresholds for q-bpMRI metrics of csPCa [5,6,9] and enable translation to clinical practice [14].

Emerging artificial intelligence (AI) deep learning (DL) reconstruction and denoising methods hold potential to substantially accelerate bpMRI and improve image quality [20,21,22]. However, their clinical utilization for quantitative mapping is hindered by undetermined biases across vendor AI/DL implementations that are trained on proprietary image collections with unknown acquisition parameters [23,24]. AI/DL models are highly susceptible to training set biases and necessitate rigorous optimization and verification [23,24]. In clinical settings, AI/DL-accelerated reconstruction tools are typically built on the scanner consoles as black-box pre-trained models and filters with limited options for adjustment parameters varying across MRI systems [23]. Furthermore, clinical scan acquisition parameters are often modified in real time on a per-patient basis, which is incompatible with prospective protocol standardization [14]. 

Quantitative validation of the front-end AI/DL-aided reconstruction and image processing provided by scanner vendors is thus particularly challenging and requires a reference platform compatible with the clinical workflow. According to QIBA and FDA guidelines for quantitative imaging devices [25,26,27], physical phantoms with known true parameter values supply the most practical means for assessment of mpMRI protocol accuracy and multi-system harmonization by correction of technical biases. Given the multitude of options and combinations of MRI acquisition and processing parameters, a comprehensive verification of AI/DL-aided quantitative relaxation and diffusion mapping protocols in the clinical environment requires an independent mpMRI reference standard of realistic complexity.

We have recently developed a quantitative multiparametric phantom for objective bias assessment of relaxation and diffusion parameters in prostate tissue relevant ranges [28]. This realistic image reference system is fully compatible with clinical scan protocols and allows objective bias assessment and correction independent of AI/DL protocol parameters and vendor algorithms. This work demonstrates the use of the mp-phantom for quantitative evaluation of biases from AI/DL-aided prostate q-bpMRI acquisition protocols and fit models. The purpose of this study was to assess acquisition protocol biases versus the added contribution of AI/DL reconstruction methods to enable quantitative T_2_ and ADC harmonization across multiple vendor platforms.

## 2. Materials and Methods

### 2.1. AI/DL-Accelerated Quantitative T_2_ and ADC Mapping Protocols

The vendor-provided prototype artificial-intelligence (AI) deep-learning (DL) reconstruction and denoising methods [20,21] were implemented for prostate apparent diffusion coefficient (ADC) and transverse relaxation time (T_2_) mapping on 3T Vida (Sys1: Siemens, Erlangen, Germany) and 3T Ingenia (Sys2: Philips, Best, The Netherlands) clinical scanners. According to vendor-provided descriptions, both methods have used the image-trained AI/DL network models for undersampled compressed sensing reconstruction. The total acceleration was a factor of six with system-default denoising, super-resolution (sr), or partial-Fourier (pf) settings. For multi-echo spin-echo (MESE) [29] or echo-planer-imaging (MEEPI) [12], T_2_ mapping used five echo-times (TE) over the range recommended in the literature (Table 1). For ADC mapping, diffusion weighted imaging (DWI), four b-values (with-averaging) were acquired as in institutional SOC practice (Table 1).

### 2.2. Patient Studies

The prospective, single-center study was IRB-approved and HIPAA-compliant. Informed consent was obtained from all participants. The AI/DL-accelerated protocols were added to SOC exams for six patients (enrolled June–December 2024) on active surveillance for PCa with lesion pathologic grade initially obtained by needle biopsy [3]. Four patients had MR visible lesions (two PIRADS 4 and two PIRADS 5) with biopsy Gleason 3 + 3. The clinical SOC1/SOC2 scans (45 min) included about 15 min of bi-parametric (T2-weighted and 4b-value DWI) acquisitions (Table 1) [30]. T_2_-mapping was not a part of SOC and was only included for AI/DL-aided acquisitions. The reconstructed voxel sizes for AI/DL images were matched to interpolated SOC values. Two raters (a clinical radiologist with 18 years and a radiology fellow with 7 years of experience) independently qualitatively compared [31,32] the SOC T2w, high-b DWI and ADC images to the corresponding AI/DL outputs for diagnostic quality (capsule demarcation and zonal anatomy contrast), resolution, signal-to-noise-ratio (SNR) and distortion using four-point Likert-like scale (as 4: better, 3: similar, 2: worse/acceptable or 1: worse/nondiagnostic). The EPI distortion was assessed visually with respect to the SOC T2w images. Lesion SNR was evaluated by visual inspection with respect to background image noise, and resolution was assessed from visual conspicuity of prostate boundaries. The overall performance for individual contrasts was summarized by median scores, and agreement between raters was measured by correlation coefficient (R) and individual score difference range.

### 2.3. Phantom Measurements

A previously developed multiparametric phantom consisting of four physical layers of hydrogels providing tissue-mimicking ADC, kurtosis (K), and T_2_ values (Supplementary Figure S1B) was used as a reference with relaxation and diffusion parameters pre-calibrated from 8-TE TSE-MESE and 8-b EPI-DWI in a high-SNR head coil as described [28]. After thermalizing overnight, the phantom was scanned at ambient temperatures (Ts = 21.0–23.5 °C) for each patient examination with patient-specific acquisition settings. Phantom temperature (±0.5 °C) was measured using NIST ADC calibration for water and 20% polyvinyl pyrrolidone (PVP20) internal standards (Supplementary Table S1) included in the jar with the multiparametric tissue mimics [28]. T_2_ and ADC values derived from AI/DL-accelerated protocols were compared to the reference values (Supplementary Figures S1 and S2) for bias estimation. Observed minor ambient temperature variation for phantom scans ensured a limited contribution to measured protocol biases. The diffusion kurtosis (DK) model [16] was used for the diffusion ADC reference standard, and mono-exponential T_2_ relaxation [11] for T_2_ reference values. These models have appropriately represented in vivo T2w and DWI signal dependence on TE and b-value ([28], Supplementary Figure S1). A single calibration reference was used for analysis of all clinical protocols. The stability of ADC(DK) reference values [28] was checked with a repeated calibration scan (Supplementary Table S2) and appeared to be within the measurement uncertainty limits (±0.05 µm^2^/ms). 

### 2.4. Quantitative Bi-Parametric (bp) MRI Analysis

ADC(4b) and MESE-T_2_ maps were reconstructed on the scanners, while MEEPI-T_2_ ADC(2b) (b = 100 s/mm^2^ and b = 800 s/mm^2^), and ADC(DK) were derived off-line using a mono-exponential model fit of voxel log-signal intensity as a function of TE and b-value, respectively. To mimic clinical workflow, no off-line image normalization, noise reduction, or artifact removal was applied.

The T_2_ MEEPI mapping [12] was performed according to the mono-exponential model:

(1) $$log(\frac{S\left(TE\right)}{S_{0}})=-\frac{TE}{T_{2}}$$

as a function of echo-time (*TE*). The ADC(2b) fit model was:

(2) $${log(S}_{b800}/S_{b100})=-\Delta b\times ADC$$

where *S_b_*_800_ and *S_b_*_100_ are the signal intensities with diffusion weighting factor, *b* = 800 and 100 (s/mm^2^). The reference diffusion kurtosis model for ADC(DK) was:

(3) $${log(S}_{b}/S_{0})=-b\times ADC+K\times (b\times ADC{)}^{2}/6$$

where *K* is diffusion kurtosis, *S_b_* and *S*_0_ are the signal intensities with and without diffusion weighting. All data analysis utilized MATLAB R2019b (Mathworks, Natick, MA, USA).

### 2.5. Phantom Metrics and Protocol Bias Measurement

Phantom T_2_ and ADC parameter histograms (Supplementary Figure S2) were generated by manually placing 50 × 15 mm^2^ rectangular regions-of-interest (ROIs) on the middle slice of the parametric maps (away from vial edges). Bin sizes for phantom T_2_ and ADC histogram were set to 3 ms and 0.03 µm^2^/ms, respectively. Measurement errors for histogram peak mean and half-width (HW) were half of the bin sizes. The protocol SNR was assessed from the ratio of the phantom histogram peak maximum to its HW (precision/uncertainty). Bias was quantified as the deviation of the measured mean parameter values from phantom reference values [26,27]:

Bias (*T*_2_
*or ADC*) = (*T*_2_
*or ADC*) − reference (*T*_2_
*or ADC*) (4)

The phantom bias dependence on measured parameter value for patient AI/DL-aided quantitative *T*_2_ and *ADC* mapping protocols on the same system (termed “repeats”) was fit to a common regression model (constant for *ADC* and quadratic for *T*_2_). The bias correction was performed by subtracting the fit bias from the measured metric value for tissue mimics. The agreement among protocols was assessed by standard deviation (SD), and accuracy was assessed by average deviation from reference values before and after correction. The level of significance (*p* < 0.05) of correction impact on *T*_2_ and *ADC* metrics in phantoms was tested by one-way ANOVA comparison of mean values for tissue mimics before and after bias correction. 

### 2.6. Quantitative Lesion Metrics Harmonization

Lesion ROIs were manually traced on a single slice of each patient’s ADC(4b) maps to avoid artifacts and partial volume. The same ROI was applied for all ADC and T_2_ measurements of a patient. Bin sizes for patient lesion T_2_ and ADC histograms were set to 5 ms and 0.05 µm^2^/ms, respectively. The lesion histogram half-width had a likely contribution from biological heterogeneity, precluding its use for precision and SNR assessment. Mean lesion T_2_ and ADC parameter values were harmonized across platforms/protocols by bias correction. The lesion bias for mean T_2_ and ADC values was corrected by subtracting the bias measured for the phantom (Equation (4)) with the patient-specific protocol. The T_2_ and ADC parameter bias was determined from the corresponding phantom fit bias value for the measured mean lesion value. The phantom-derived bias corrections were compared for measured lesion ADC dependence on b-range. The correction (harmonization) efficacy was assessed by average residual differences across lesions for corrected ADC(2b) and ADC(4b) versus measured ADC(DK).

## 3. Results

### 3.1. Qualitative Assessment

The AI/DL accelerated four-b DWI and five-TE T_2_ scans (Table 1) took less than 6 min on each MRI platform (less than half of the corresponding standard-of-care bpMRI examination time). Among SOC and AI/DL scan protocols, b-value sets were consistent while other acquisition parameters varied (Table 1). Acquired AI/DL T2w in-plane resolution (2 × 2 mm^2^) and high-b-averages (8) for DWI were lower than SOC1 (0.5 × 0.6 mm^2^ and 20) or SOC2 (0.4 × 0.7 mm^2^ and 16). TR parameter varied most between patient scan protocols on Sys1, and for AI-accelerated T_2_ and ADC on Sys2. Different through-slice resolutions (4 mm and 3 mm) were tested for AI/DL-aided EPI DWI. DL reconstruction protocol on Sys1 was compatible only with EPI, hence MEEPI was implemented for quantitative T_2_-mapping for patient scans on this system. All SOC scans had adequate diagnostic quality, resolution, and SNR, and an acceptable level of distortion. The same (factor of 6) acceleration and number of b-averages without DL/AI-aided denoising would yield an unacceptable level of noise and distortion in SOC DWI scans. 

**Figure 1:**
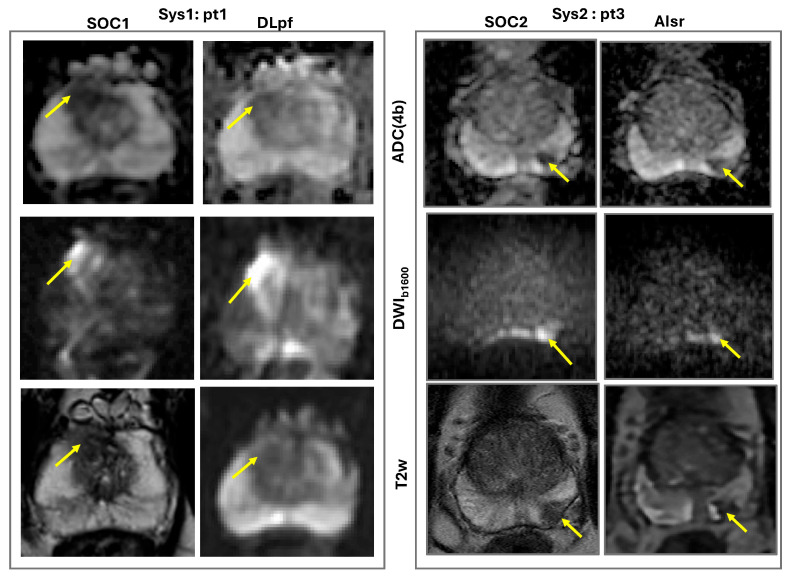
Gray-scale images qualitatively compare examples of standard of care (SOC) bi-parametric (bp)MRI versus deep-learning partial-Fourier (DLpf) and artificial-intelligence super-resolution (AIsr) from two different vendor 3T systems (Sys1 and Sys2) in two patients (pt1 and pt3) with PIRADS 4 lesions (arrows) in the right anterior mid-gland (pt1, left) and left posterior apex (pt3, right) peripheral zone.

Four MRI visible lesions were subjectively evaluated by two raters on a 4-pt Likert-like scale for AI/DL-aided reconstruction protocols in comparison to SOC for diagnostic quality, contrast, SNR, resolution, and distortion (Table 2). The overall interrater agreement was good (R = 0.78) with a maximum individual score deviation of ±1. In general, both raters confirmed acceptable diagnostic quality for AI/DL abbreviated protocols, similar to SOC (median Likert-like scores 2–3 for Sys1 and 3–4 for Sys2 patient scans), although individual sequence performance varied. Overall, lesion DWI and T2w contrast features of adequate (acceptable or similar) diagnostic quality were perceived on DL/AI versus SOC bpMRI (Figure 1, Table 2), except for T2w (DLpf-EPI) of Sys1-pt2 with high EPI-distortions. Median performance scores were higher for Sys2 patients and for T2w images derived from AIsr-MESE versus DLpf-MEEPI. 

**Figure 2:**
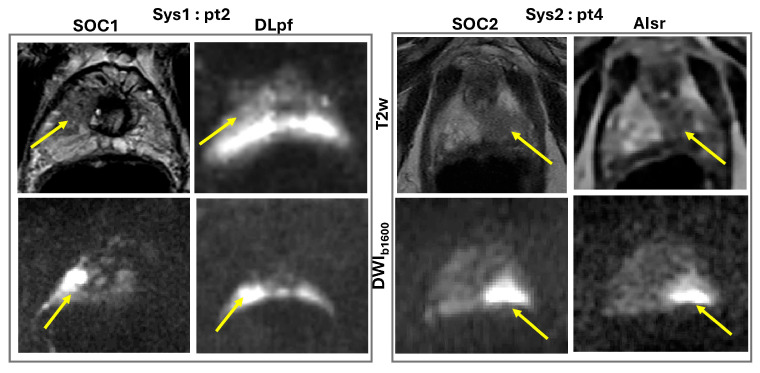
Different sensitivity to susceptibility distortions for standard of care (SOC1/SOC2) versus DL/AI accelerated bi-parametric (bp)MRI on two 3T systems (Sys1 and Sys2) for two patients (pt) with peripheral zone PIRADS 5 lesions (arrows) in the right apex (pt2) and left apex (pt4).

For DWI_b1600_ and ADC, both DLpf (Sys1) and AIsr (Sys2) methods showed similar or improved SNR compared to SOC for 4 mm slices (scores of 3 and 4). SNR was lower, and acceptable for 3 mm slices, but the resolution of AIsr (Sys2) reconstruction was always better than SOC. Interestingly, the DLpf (Sys1) protocol appeared to be more susceptible to EPI-distortions compared to AIsr (Sys2) reconstruction, in the presence of rectal air (Figure 2). DLpf (Sys1) MEEPI protocol showed generally lower performance for T2w images compared to SOC in resolution, SNR, and distortion. AIsr (Sys2) method was compatible with MESE protocol, leading to similar or better T2w SNR, resolution, and distortion versus SOC images, and would be preferred over T2w MEEPI (Table 2, Figure 2).

### 3.2. Quantitative Assessment

**Figure 3:**
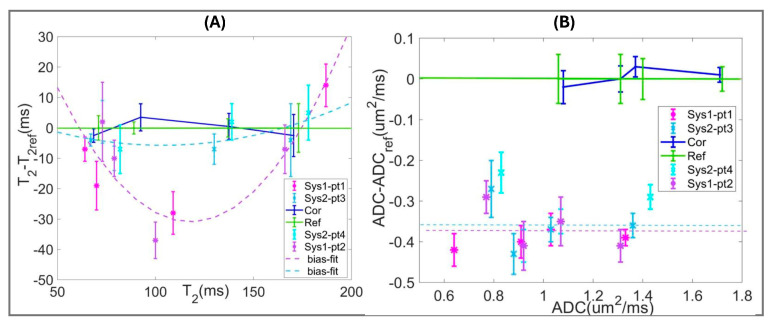
Quantitative bias assessment for multiparametric phantom scanned with four DL/AI accelerated bpMRI protocols (“Sys1-pt1”, “Sys1-pt2”, “Sys2-pt3”, “Sys2-pt4”, color-coded in the legends) used for patients (pt) with MRI-visible lesions (Table 1). The plots summarize mean bias (asterisks) versus reference values for T_2_ (**A**) and ADC (**B**). Error bars correspond to the phantom ROI histogram peak half-width (HW, see Supplementary Figure S2). Dashed lines are fits for the measured biases: constant for ADC [Sys1-pt1,2 and Sys2-pt3,4] = [0.38 and 0.36] µm^2^/ms; and quadratic for T_2_ (Sys1-MEEPI and Sys2-MESE bias fit coefficients listed in Supplementary Figure S3). Solid blue lines indicate residual parameter differences between protocols after corresponding bias correction (“Cor”). Solid green line marks zero bias/difference with error-bars indicating the reference (“Ref”) histogram HW (see Supplementary Figure S1).

Figure 3 illustrates an objective quantitative assessment of scan-protocol bias for AI/DL-accelerated acquisitions (used for four MR-visible lesions) with respect to the DK phantom reference (Supplementary Figure S2). The bias measured from the shift in DL/AI-aided protocol histogram peaks from the reference peaks (Supplementary Figure S2, Equation (4)) for tissue mimics is summarized in Table 3. The parameter measurements for additional protocols before and after bias correction are detailed in Supplementary Tables S3 and S4.

Over the reference T_2_ range (70–170 ms) (Figure 3A), the observed AI-aided MESE T_2_ protocol bias for Sys2 (pt3 and pt4) is relatively constant with histogram peaks mostly shifting to lower values by −7 ms to −5 ms, except for a +5 ms shift for Atr-mimic. In contrast, DL-aided MEEPI T_2_ protocol bias for Sys1 (pt1 and pt2) is markedly nonlinear with increasing negative shifts from −10 ms to −37 ms for nTZ and nPZ mimics and positive +14 ms for Atr-mimic. The comparative analysis of MEEPI protocols implemented alongside MESE on Sys2 (where AIsr reconstruction was compatible with both EPI and SE acquisition) confirmed similarity of nonlinear T_2_ bias trends with Sys1 (Supplementary Figure S3). These results reflect T_2_ bias dependence on SE versus EPI acquisition protocol variants for T_2_ mapping (rather than AI/DL reconstruction). Finite differences observed between protocol repeats on the same system (Figure 3A, Supplementary Figure S3) are within measurement uncertainties (error bars) and likely due to variations in acquisition parameters (e.g., TR, Table 1) and scanner room temperatures (Table 3). Compared to uncorrected values (Supplementary Table S3), bias correction minimizes differences to reference T_2_ values (Supplementary Table S4), most effectively for nTZ and nPZ mimics. It also reduces variability for phantom T_2_ values across protocols, as is evident from a 5 to 6-fold reduction in SD for T_2_ of nPZ and Atr mimics (Supplementary Table S4). 

ADC(4b) model bias (Figure 3B) is consistent between AI/DL reconstruction protocols and system repeats, and manifests primarily as a shift to lower ADC versus reference DK values (similar to Supplementary Figure S1B with regular reconstruction). This result is highly suggestive of bias stemming from the ADC fit model rather than AI/DL-aided reconstruction. Minor variability between ADC protocol repeats is mainly due to temperature and EPI distortion (e.g., evident for phantom Sys1-pt1 (DLpf) protocol at ADC = 0.75 µm^2^/ms). Different biases would be observed for the ADC(2b) model (Supplementary Table S3), leading to increased variability of measured values across protocols. Except for Atr-mimic (*p* = 0.62), bias correction significantly (*p* < 0.015) reduced T_2_ and ADC parameter differences both between protocols and with respect to reference values for the phantom materials (Figure 3, blue, Supplementary Table S4), down to residual deviations due to temperature dependence of measured parameters.

The precision (histogram peak HW) and SNR are apparently similar to those of the DK phantom reference (Table 3, Figure 3B) for ADC(4b) of both Sys2-pt3 (AIsr) and Sys1-pt2 (DLpf) protocols (HW = 0.04–0.07 µm^2^/ms, with 3 mm slices). The precision is marginally better than the reference for Sys1-pt1 and Sys2-pt4 protocols (HW = 0.02–0.05 µm^2^/ms, with 4 mm slices), suggesting overall efficient AI/DL-aided denoising for EPI DWI acquisition. The SNR of both Sys1-DLpf (5TE-MEEPI) and Sys2-AIsr (5TE-MESE) T_2_ mapping protocols is lower than that of the reference (Table 3, Figure 3A). Phantom T_2_ precision is marginally better for Sys2 (HW = 2–6 ms) than for Sys1 protocols (HW = 4–12 ms), excepting the “Atr” mimic (long T_2_ peak). Lower SNR and large nonlinear bias are observed for the Sys1 T_2_ (MEEPI) protocol, apparently due to EPI distortion (compared to distortion-free MESE).

It is noteworthy that AI/DL-aided reconstruction for phantom scans mainly affected precision (HW) and SNR, while absolute bias was due to acquisition protocol (T_2_ MEEPI versus MESE and TE-range) or fit-model (ADC b-range).

### 3.3. Quantitative bpMRI Harmonization

**Figure 4:**
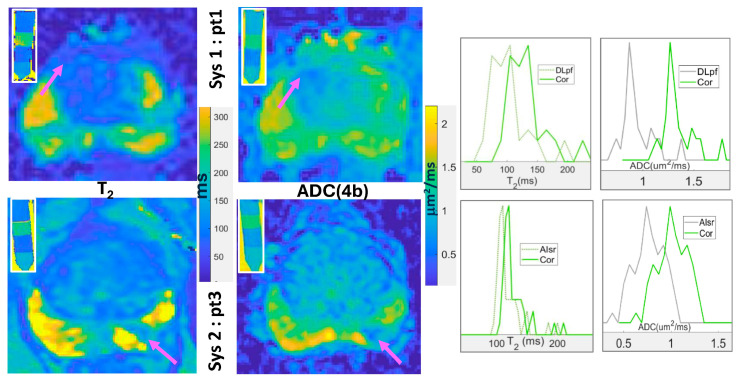
Bi-parametric Sys1-DLpf (top row) and Sys2-AIsr (bottom row) maps (color) and lesion histograms for T_2_ and ADC(4b) (bin sizes: 5 ms and 0.05 µm^2^/ms) generated for two patients (pt1 and pt3) with PIRADS 4 lesions (arrows) scanned on different clinical 3T MRIs (Sys1, Sys2). Color bars indicate quantitative parameter scales. The inserts show corresponding maps for the multiparametric phantom used to assess protocol biases (Figure 3) that are corrected (“Cor”) to harmonize T_2_ and ADC values for lesion histograms (right).

The use of measured protocol biases (Figure 3, Table 3) for harmonization of lesion T_2_ and ADC of PCa patients scanned on different MR platforms is illustrated in Figure 4. The lesion SNR tends to be visually higher on ADC and T_2_ maps compared to individual b-value and TE weighted images (Figure 1), likely owing to multiple images used for parametric mapping. Bias correction implemented by subtracting the correction factors measured for the phantom is manifesting in shifts to higher values both for T_2_ and ADC lesion histograms (Figure 4, green). Larger shifts had to be applied for bias correction of ADC(4b) (Sys1-pt1: 0.38 µm^2^/ms & Sys2-pt3: 0.36 µm^2^/ms) and T_2_-MEEPI (Sys1-pt1: 30 ms) compared to T_2_-MESE (Sys2-pt3: 10 ms). The lesion T_2_ and ADC(4b) values harmonized to the DK reference across protocols and scan systems are listed in Table 4. The reference parameter ranges in the phantom (Figure 4, inserts) were apparently adequate to accommodate measured lesion values but would need to be expanded for normal (non-lesion) tissue bias correction (e.g., higher T_2_ and ADC values are needed for atrophy mimics).

**Figure 5:**
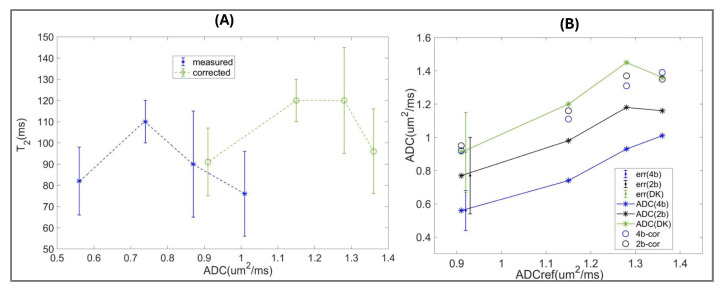
(**A**) Average lesion T_2_ versus ADC values are plotted before (blue asterisks) and after bias correction (green circles). The error bars correspond to the T_2_ histogram half-width (HW). (**B**) Efficiency of ADC model harmonization (4b, 2b versus DK) is illustrated for four lesions by alignment of measured (green asterisks) and corrected (blue and black circles) ADC values. The data are color-coded in the legend. The error bars show the representative ADC histogram HW for the first lesion. The lines in (**A**,**B**) connect the measured data (symbols) for visual guidance.

After bias correction, the harmonized T_2_ and ADC values for lesion histograms (Table 4) can be quantitatively compared across patient scans independent of acquisition protocol. Similarly to phantom results, the main differences (Δ) in lesion T_2_ values before and after correction are associated with MEEPI (ΔT_2_ = 20–30 ms) versus MESE (ΔT_2_ = 9–10 ms) protocols, while for ADC(4b) bias is primarily determined by the fit model (ΔADC = 0.36–0.38 µm^2^/ms, Figure 5A). The bias correction results in equal T_2_ values for pt1 and pt3 lesions and confirms pt4 lesion as having relatively low ADC(DK) and T_2_ values, suggesting a higher likelihood of clinically significant PCa. The HW of lesion T_2_ (Table 4) for Sys2 (pt3 and pt4) is about half of Sys1 (pt1 and pt2), likely reflecting better SNR and lower distortion for MESE versus MEEPI acquisition. The HW for ADC(4b) is apparently correlated to the DWI slice width doubling for 3 mm versus 4 mm slices, both for Sys1 (DLpf) and Sys2 (AIsr) protocols. This is likely related to intrinsic limits of the corresponding DL/AI denoising models.

Phantom multiexponential diffusion materials allow evaluation of ADC dependence on b-value (Supplementary Figure S1). Consistent increase in ADC(DK) versus AIsr-ADC(2b) versus ADC(4b) is observed for phantom (Supplementary Figure S1, Tables S3 and S4) and PCa patient lesions (Figure 5B). The bias measured for mp-phantom (Figure 3B) closely represents differences between ADC models in vivo (Figure 5B, circles versus asterisks). The average difference between ADC(4b) and ADC(2b) fit models with measured lesion ADC(DK) is reduced 5–6-fold by correction, down to measurement uncertainty levels (from 0.42 and 0.21 µm^2^/ms to 0.07 and 0.04 µm^2^/ms). For studies that do not include high-b acquisition, ADC(2b) values derived in a lower-b-range would require harmonization with values derived using high-b ADC protocols (mean bias of 0.21 µm^2^/ms, Figure 5). Without bias correction, the use of low-b ADC(2b) to synthesize high-b DWI (Supplementary Figure S1A) would also artefactually decrease SNR for tissue with true kurtotic diffusion restriction, lowering the sensitivity of lesion detection. The HW for ADC(4b) lesion histograms is about half of ADC(2b) or ADC(DK). Thus, in practice, the 4b-fit may be preferred for the best achievable ADC SNR, and derived ADC(4b) values can be aligned with DK reference values by retrospective bias correction (as illustrated in this work). Note that proposed harmonization cannot be achieved with a mono-exponential diffusion references (e.g., PVP) that have no ADC dependence on b-value or with a reference tissue that has no or low kurtosis (e.g., body fluids).

## 4. Discussion

AI/DL accelerated acquisition [20,21] is desired to enable clinical implementation of quantitative bi-parametric MRI for improved specificity of clinically significant PCa risk stratification [6,8,9,14]. The goal of this study was to build a clinically viable validation workflow for AI/DL-aided reconstruction and denoising of prostate q-bpMRI and harmonize derived T_2_ and ADC values across different vendor platforms and acquisition protocols. Our study demonstrated the feasibility of flexible multi-system harmonization of q-bpMRI using AI/DL accelerated acquisitions by employing a multiparametric reference platform. This realistic-complexity platform allows direct quantitative testing of vendor-provided protocols with arbitrary settings for *in vivo* acquisition parameters with respect to the reference T_2_ and ADC values. The implemented workflow enabled assessment of individual bias contributions from acquisition protocols versus DL/AI-aided reconstruction versus parametric fit models. The performed phantom reference-based harmonization follows FDA guidelines for quantitative imaging devices [26] that are being adopted by quantitative imaging modalities [33,34]. The described method is generally more practical for prospective clinical application on a per-patient/per-protocol basis compared to traveling volunteer or reference tissue harmonization approaches [10,35].

Our study confirmed the benefits of AI/DL accelerated bpMRI for prostate imaging [20,21,32,36] that achieved two-fold acquisition time saving with acceptable diagnostic quality (comparable to SOC) on two clinical 3T scanners. Importantly, the tested vendor-provided AI/DL tools improved SNR or resolution for EPI-DWI without added bias and were compatible with EPI acquisition on both vendor systems. The qualitative tests for ADC and DWI confirmed higher efficiency of AI/DL-aided reconstruction for EPI-DWI denoising and of the AIsr method for improved resolution and reduced distortion, outperforming SOC. However, improved denoising was limited by slice thickness (to 4 mm), while the DLpf method appeared more susceptible to EPI distortion. For T_2_ mapping, the AIsr combination with MESE provided the best performance. These results are consistent with other prostate MRI studies that reported qualitative evaluations of AI/DL-aided reconstruction and denoising [20,21,22,32,36].

Much of the current research on multi-system harmonization of bi-parametric prostate MRI has been focused on downstream lesion segmentation and classification analysis in retrospective studies [37,38,39]. Harmonized quantitative metrics are a prerequisite for advanced predictive modeling using radiomics [40,41,42] and improved yield of multicenter clinical trials [14,24,27,37]. In the absence of reference standards and prospective acquisition protocol standardization, such studies largely rely on statistical and machine learning methods for retrospective data normalization [38,41,42]. Upstream elimination of large technical biases by reference standard harmonization for multiple acquisition conditions [26,33,34] improves generalization and reduces the numbers required for training downstream statistical harmonization models [37,38,41,42]. While QIBA recommendations for prospective standardization of acquisition protocol parameters [14,25,27,43] are being implemented in multicenter clinical trials to help minimize cross-platform biases in longitudinal studies (e.g., useful for treatment response assessment), their translation to SOC clinical practice remains challenging, particularly for diagnostic applications [14,24,27,43]. Cross-platform standardization of vendor-provided AI/DL-aided reconstruction based on proprietary algorithms and models [23] introduces an additional level of complexity. Regulatory acceptance of harmonized quantitative biomarkers in prostate cancer management necessitates validation of all components of the AI/DL-aided acquisition and analysis workflow [26,27]. The previous in vivo studies of AI/DL-accelerated acquisition protocols primarily conducted qualitative assessment for patients scanned on a single vendor platform [22,32,36] or utilized a single-parameter phantom that required a dedicated evaluation protocol [44].

The present prospective study implements the recommended harmonization approach [26,27,33,34] by combining qualitative assessment of AI/DL-aided q-bpMRI acquisition with quantitative bias measurement using a multiparametric reference phantom compatible with clinical protocols across different vendor systems. Multiparametric reference [28] allows objective testing of the SOC versus AI/DL reconstruction enhancements [23,26]. The implemented workflow enabled characterization of two distinct sources of biases in derived quantitative T_2_ and ADC parameters: (1) scan and reconstruction protocol settings and (2) fit model. Using the reference phantom, we measured and corrected the protocol-dependent biases in T_2_ and ADC values to bring them on a comparable scale for MRI visible lesions. Nonlinear bias was detected for T_2_-MEEPI versus nearly constant for T_2_-MESE, while ADC bias was due to b-range dependence in the presence of diffusion kurtosis. Importantly, our results suggest that reference tissue normalization based on body fluids [10] could be generally inadequate for prostate ADC and T_2_ across system protocols, when reference values have different dependence on acquisition parameters compared to PCa lesions due to tissue-dependent diffusion kurtosis or nonlinear T_2_ biases. Measurement accuracy for reference tissue (e.g., bladder [10] or muscle) also depends on anatomic coverage, which may be limited for reduced field-of-view acquisitions [31]. Demonstrated consistency of bias dependences in phantom and lesions, and for system protocol repeats, suggests that practical bias assessments and protocol optimizations are possible both prior to and after patient scans using mp-phantom for the ranges of target clinical acquisition parameters.

Our study had several limitations. The tests were performed on two systems with a small number of MR-visible low-grade lesions and a two-reader assessment. This may potentially increase the variability of qualitative scores for subjective evaluation, although we observed good inter-reader agreement and consistency with quantitative phantom SNR evaluation. Larger population, multi-system, multi-reader studies with correlation to clinical outcomes (including high and low PCa grades) are needed to ascertain the added benefit of the AI/DL-accelerated q-bpMRI protocols for PCa patient management [14,23,36]. The AI/DL parameters were not optimized, and default settings were used provided by vendor implementations for a realistic illustration of the clinical application. Further optimization is possible [20,21,31,36] to increase MESE T_2_ resolution, achieve compatibility with non-EPI acquisition, and optimize EPI-distortion correction.

Harmonization was based on the correction of additive bias for mean ROI metrics, which do not affect voxel-based image texture features and histograms used for radiomics analysis [40,41,42]. For such applications, the corrected T_2_ and ADC maps could be utilized to generate synthetic DWI and T2w images harmonized by using the same b-values and TEs. For more accurate bias measurement and correction, there is also a need to increase the reference range for ADC and T_2_ of mp-phantom to accommodate values for non-lesion tissue and perform temperature calibration studies for reference values within the scanner room temperature ranges [45,46]. These calibrations can be performed once and used prospectively to improve the precision of bias measurement for clinical protocols at ambient scanner room temperatures. Assessment of T_2_ biases due to varying protocol TR could also be improved by including phantom materials with tissue-mimicking T_1_ values. Finally, additional multi-system, multi-TE MESE and multi-b DWI *in vivo* studies are needed to confirm the consistency and accuracy of T_2_(TE) protocol biases and ADC b-range biases detected with the phantom.

## 5. Conclusions

The developed protocol enables harmonization of the vendor-provided AI/DL-accelerated acquisition options to establish credibility and streamline implementation of prostate q-bpMRI protocols in a multi-vendor clinical environment. AI/DL-aided reconstruction reduces acquisition time with improved SNR and resolution versus the SOC protocol. A multiparametric phantom allows assessment of biases directly for clinical scan protocols with arbitrary acquisition settings on a per-patient basis. The observed biases were due to acquisition parameters for T_2_-mapping and the b-range model fit for ADC-mapping. No added biases were detected from AI/DL reconstruction and denoising. The proposed workflow facilitates improved reproducibility and accuracy of AI/DL-aided quantitative ADC and T_2_ mapping for prostate patients in the SOC setting.

## Figures and Tables

**Table 1:** Essential acquisition protocol parameters.

Protocol	TE (ms)	TR (s)	b[nav] (s/mm^2^)	Acquired Voxel (mm^3^)	Scan Duration (min)
**Sys1:**					
**SOC1-T2w**	107	* 9.2/4.8	NA	0.5 × 0.6 × 3	6:00
**SOC1-DWI**	91	**4.4/4.8**	0[1], 100[1], 800[3], 1600[20]	1.75 × 1.75 × 4	8:50
**DLpf-T2 (MEEPI)**	40, 70, 100, 130, 160	**5.5/6.8**	NA	2 × 2 × 3 (off-line map)	2:10
**DLpf-ADC**	80	5.5	0[1], 100[1], 800[2], 1600[8]	2 × 2 × **4/3**	3:30
**Sys2:**					
**SOC2-T2w**	110	4.4	NA	0.4 × 0.7 × 3	5:00
**SOC2-DWI**	77	7.2	0[2], 100[2], 800[4], 1600[16]	2.2 × 2.3 × 4	8:20
**AIsr-T2 (MESE)**	25, 65, 105, 145, 185	**8/12.2**	NA	2 × 2.3 × 3	1:45
**AIsr-ADC**	77	**3.9/5.4**	0[1], 100[1], 800[2], 1600[8]	2 × 2 × **3/4**	4:00

* Parameters in bold changed between patient scans; TE: echo time; TR: repeat time; [nav]: number of averages per b-value; Sys1: Siemens Vida; Sys2: Philips Ingenia; SOC: standard of care; DLpf: deep-learning partial-Fourier; AIsr: artificial-intelligence super-resolution; MEEPI: multi-echo echo-planer imaging; MESE: multi-echo spin-echo.

**Table 2:** Likert-like scores (rater 1/rater 2) for qualitative comparison of SOC versus AI/DL-accelerated bpMRI for patients with MR-visible lesions.

Image	Eval. Criteria	Sys1-pt1 (PIRADS 4)	Sys1-pt2 (PIRADS 5)	Sys2-pt3 (PIRADS 4)	Sys2-pt4 (PIRADS 5)
**ADC**	**Dx quality**	3/3	2/3	3/4	3/3
	**distortion**	3/3	2/2	4/3	4/4
	**resolution**	3/2	2/2	4/4	4/4
	**SNR**	4/3	2/2	3/4	4/4
	**median**	3/3	2/2	3.5/4	4/4
**DWI_b1600_**	**Dx quality**	3/3	2/2	3/3	4/3
	**distortion**	3/3	2/2	4/3	4/4
	**resolution**	2/2	2/2	4/3	3/4
	**SNR**	4/4	2/2	2/3	4/4
	**median**	3/3	2/2	3.5/3	4/4
**T2w**	**Dx quality**	2/2	1/1	3/3	4/3
	**distortion**	2/3	1/2	3/3	3/3
	**resolution**	2/2	2/2	2/2	3/2
	**SNR**	3/2	2/2	4/4	4/4
	**median**	2/2	1.5/2	3/3	3.5/3

Sys-pt: system—patient; ADC: apparent diffusion coefficient; SNR: signal-to-noise ratio; DWI_b1600_: diffusion weighted image for b = 1600 s/mm^2^; T2w: T2 weighted image; Dx: diagnostic.

**Table 3:** T_2_ and ADC parameters (histogram mean and half-width) measured for bp-phantom tissue mimics using patient-specific AI/DL-aided Sys1 and Sys2 protocols and reference scans.

Protocol (Ts ± 0.5 °C)	Parameter Mean [HW]	GS7	nTZ	nPZ	Atr
**DKref (22.0)**	**ADC ± 0.015 (mm^2^/ms)**	1.06 [0.06]	1.33 [0.06]	1.42 [0.04]	1.72 [0.04]
**Sys1-pt1 (21.4)**		0.64 [0.04]	0.91 [0.04]	1.03 [0.02]	1.33 [0.02]
**Sys1-pt2 (21.1)**		0.77 [0.05]	0.92 [0.06]	1.07 [0.06]	1.31 [0.04]
**Sys2-pt3 (21.0)**		0.79 [0.07]	0.88 [0.06]	1.03 [0.04]	1.36 [0.04]
**Sys2-pt4 (23.5)**		0.83 [0.05]	0.92 [0.04]	1.07 [0.03]	1.43 [0.03]
**T2ref (22.0)**	**T_2_ ± 1.5 (ms)**	71 [4]	89 [2]	137 [3]	173 [8]
**Sys1-pt1 (21.4)**		64 [4]	70 [8]	109 [7]	187 [7]
**Sys1-pt2 (21.1)**		73 [12]	79 [6]	100 [6]	166 [8]
**Sys2-pt3 (21.0)**		67 [2]	79 [4]	130 [5]	169 [10]
**Sys2-pt4 (23.5)**		73 [4]	82 [5]	139 [6]	178 [9]

Ts: phantom scan temperature; HW: half-width; GS7: Gleason7, TZ: transition-zone, PZ: peripheral-zone, Atr: atrophy tissue mimics; DKref: diffusion kurtosis reference; Sys-pt: system—patient protocol.

**Table 4:** Measured and corrected T_2_ and ADC (mean and half-width) for lesion histograms.

Parameter Mean [HW]	Sys1-pt1	Sys1-pt2	Sys2-pt3	Sys2-pt4
**T2meas ± 3 (ms)**	90 [25]	76 [20]	110 [10]	82 [16]
**T2cor ± 3 (ms)**	120 [25]	96 [20]	120 [10]	91 [16]
**ADC(4b)meas ± 0.03 (mm^2^/ms)**	0.93 [0.09]	1.01 [0.18]	0.75 [0.24]	0.56 [0.12]
**ADC(4b)cor ± 0.03 (mm^2^/ms)**	1.31 [0.09]	1.39 [0.18]	1.11 [0.24]	0.92 [0.12]
**ADC(2b)meas ± 0.03 (mm^2^/ms)**	1.18 [0.16]	1.16 [0.31]	0.98 [0.36]	0.77 [0.23]
**ADC(2b)cor ± 0.03 (mm^2^/ms)**	1.37 [0.16]	1.35 [0.31]	1.16 [0.36]	0.95 [0.23]
**ADC(DK)meas ± 0.03 (mm^2^/ms)**	1.45 [0.2]	1.36 [0.44]	1.2 [0.38]	0.91 [0.24]

HW: half-width; Sys-pt: system—patient; meas: measured; cor: corrected; ADC(4b): four b-value ADC fit; ADC(2b): two b-value ADC fit; ADC(DK): diffusion kurtosis ADC fit.

## Data Availability

Essential data is contained in the article and Supplementary Figures.

## Supplementary Materials

The following supporting information can be downloaded at: https://www.mdpi.com/article/10.3390/s25185858/s1, Figure S1: (A) Example of b-range dependence for different ADC fit models (color-coded in the legend) for log-DWI signal, Sb (top), and corresponding parametric maps (bottom). (B) ADC b-range dependence reproduced in the layered diffusion kurtosis (DK) reference phantom (at scan temperature Ts). The top stacked plot shows ADC calibration histograms for three fit models (color-coded in the legend) generated from the parametric maps under the plot for the ROI (bin sizes: 0.03mm^2^/ms) marked on the kurtosis (K) map. Color-bar shows common ADC (and K) scale for mono-exponential fit models using all b-values (“4b”), 2b-values (“b100b800”) and diffusion kurtosis (“DK”). The kurtosis values [K] ± 0.05 are listed next to reference (“ref”) DK histogram peaks (green). Reference peak labels mark corresponding prostate tissue mimics (GS7: Gleason7, TZ: transition-zone, PZ: peripheral-zone, Atr: atrophy. Figure S2: The stacked plots compare histograms for AI/DL-aided acquisition protocols (Table 1, color-coded in the legends) versus calibrated reference (green) of T_2_ (A, bin size 3 ms) and ADC (B, bin size 0.03 μm^2^/ms) generated from the corresponding parametric maps that share the common scales (color-bars.) Phantom scan temperatures (ts ± 0.5 °C) are listed above the ADC maps (right). Figure S3: T_2_ bias comparison for AI-accelerated MEEPI versus MESE scan protocols for multi-parametric phantom. The mean T_2_ biases measured on Sys2 with respect to reference values are plotted (blue and cyan asterisks) for two repeated MEEPI scans (TE = [25, 55, 85, 115, 145] ms, TR = 6 s, Ts = 21 °C & 23.5 °C). Error-bars correspond to the phantom ROI histogram peak half-width (HW, see Figure S2). Solid green line marks zero bias/difference with error-bars indicating the reference (“Ref”) histogram HW (see Figure S1). Dashed lines are quadratic fits for the measured biases: for Sys2 AIsr T_2_-MEEPI with fit coefficients [c2, c1, c0] = [0.004, −0.95, 37.57], and for T_2_–MESE [c2, c1, c0] = [0.002, −0.314, 10.513] (data shown in Figure 3A), as well as for Sys1 DLpf-accelerated T_2_-MEEPI [c2, c1, c0] = [0.01, −2.26, 102.23] (Figure 3A). Table S1: Measured internal standard ADC values used to derive phantom scan temperature, Ts, using NIST calibration [https://data.nist.gov/od/id/mds2-2366 accessed on 3 August 2025]. Table S2: DK phantom reference values for repeated calibration (b = 0, 200, 500, 800, 1500, 2000, 2500 s/mm^2^; Ts = 21.0 ± 0.5 °C). Table S3. ROI mean T_2_ and ADC parameters for bp-phantom tissue mimics across different scan protocols. Table S4. ROI mean T_2_ and ADC parameters for bp-phantom tissue mimics corrected using bias-fit for different scan protocols with respect to DK reference values. References [28,30,46,47] are cited in the Supplementary Materials.

## Abbreviations

The following abbreviations are used in this manuscript:

ADC	Apparent diffusion coefficient
QIBA	Quantitative Imaging Biomarker Alliance
NIST	National Institute of Standards
DWI	Diffusion weighted imaging
T2w	T2 weighted
DLpf	Deep-learning partial-Fourier
AIsr	Artificial-intelligence super-resolution
q-bpMRI	Quantitative bi-parametric MRI
SOC	Standard-of-care
MESE	Multi-Echo Spin-Echo
MEEPI	Multi-Echo echo-planar imaging
TE	Echo-time
DK	Diffusion kurtosis
HW	Half-width
Sys	system
pt	patient
PVP	polyvinylpyrrolidone
