# Supplementary material for: Cross-Scanner Harmonization of AI/DL Accelerated Quantitative Bi-Parametric Prostate MRI
Source: Sensors (Basel). 2025 Sep 19;25(18):5858. doi: 10.3390/s25185858 (PMC12473519; doi:10.3390/s25185858)
Supplement: Supplementary file 1 [file sensors-25-05858-s001.zip › sensors-3832095-supplementary.pdf]

Supplementary Figures

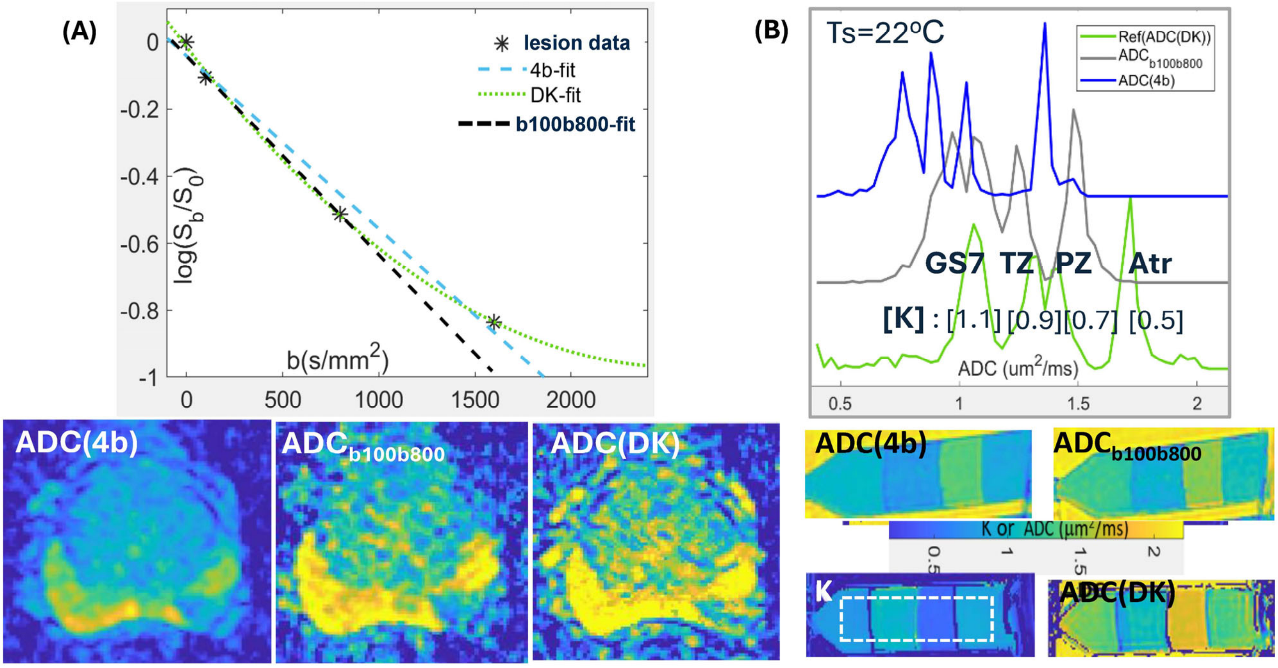

Supplementary Figure S1: (A) Example of b-range dependence for different ADC fit models (color-coded in the legend) for log-DWI signal, Sb (top), and corresponding parametric maps (bottom). (B) ADC b-range dependence reproduced in the layered diffusion kurtosis (DK) reference phantom (at scan temperature Ts). The top stacked plot shows ADC calibration histograms for three fit models (color-coded in the legend) generated from the parametric maps under the plot for the ROI (bin sizes: 0.03µm^2/ms) marked on the kurtosis (K) map. Color-bar shows common ADC (and K) scale for mono-exponential fit models using all b-values ("4b"), 2b-values ("b100b800") and diffusion kurtosis ("DK"). The kurtosis values [K]±0.05 are listed next to reference ("ref") DK histogram peaks (green). Reference peak labels mark corresponding prostate tissue mimics (GS7: Gleason7, TZ: transition-zone, PZ: peripheral-zone, Atr: atrophy.

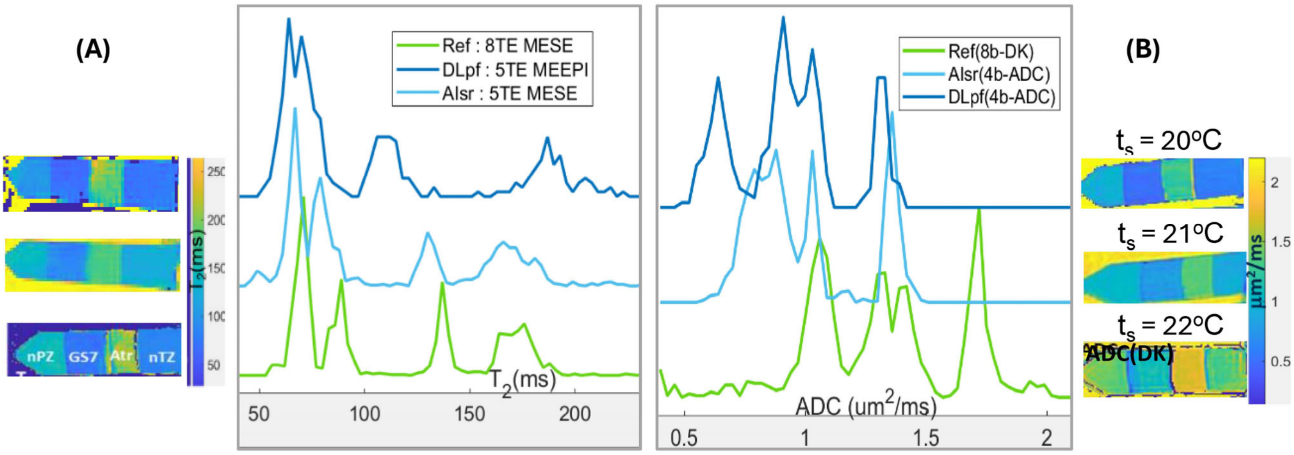

Supplementary Figure S2: The stacked plots compare histograms for AI/DL-aided acquisition protocols (Table 1, color-coded in the legends) versus calibrated reference (green) of T2 (A, bin size 3ms) and ADC (B, bin size 0.03µm^2/ms) generated from the corresponding parametric maps that share the common scales (color-bars.) Phantom scan temperatures (ts±0.5°C) are listed above the ADC maps (right).

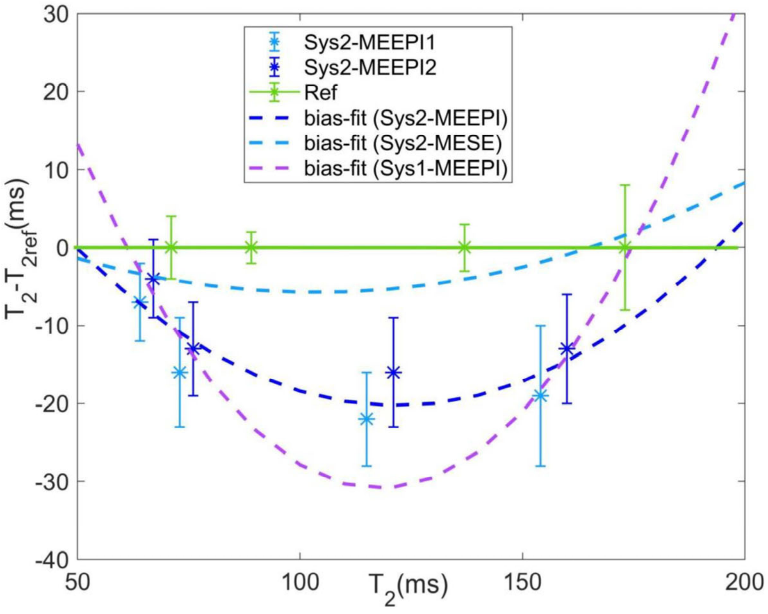

Supplementary Figure S3: T_2 bias comparison for AI-accelerated MEEPI versus MESE scan protocols for multi-parametric phantom. The mean T_2 biases measured on Sys2 with respect to reference values are plotted (blue and cyan asterisks) for two repeated MEEPI scans (TE = [25, 55, 85, 115, 145]ms, TR=6s, Ts=21°C & 23.5°C). Error-bars correspond to the phantom ROI histogram peak half-width (HW, see Sup. Fig.S2). Solid green line marks zero bias/difference with error-bars indicating the reference ("Ref") histogram HW (see Sup. Fig..S1). Dashed lines are quadratic fits for the measured biases: for Sys2 AIsr T_2-MEEPI with fit coefficients [c2, c1, c0] = [0.004, -0.95, 37.57] , and for T_2–MESE [c2, c1, c0] = [0.002, -0.314, 10.513] (data shown in Fig.3A), as well as for Sys1 DLpf-accelerated T_2-MEEPI [c2, c1, c0] = [0.01, -2.26, 102.23] (Fig.3A).

References:

1. Dariya Malyarenko, Scott D. Swanson, Jacob Richardson, Suzan Lowe, James O'Connor, Jesus E Fajardo, Yun Jiang, Shane Wells, Thomas L. Chenevert. Harmonization of AI/DL accelerated quantitative bi-parametric prostate MRI: demonstration in multi-parametric phantom and patients. 2025 ISMRM, Proceedings PP103, 5/2025, Honolulu HI, USA
2. Amouzandeh G, Chenevert TL, Swanson SD, Ross BD, Malyarenko DI: Technical note: Temperature and concentration dependence of water diffusion in polyvinylpyrrolidone solutions. Med Phys 2022; 49(5): 3325-32
3. M. Holz, S. R. Heil, and A. Sacco, Temperature-dependent self-diffusion coefficients of water and six selected molecular liquids for calibration in accurate 1H NMR PFG measurements. Phys. Chem. Chem. Phys. 2000; 2(2):4740–2.
4. Malya*ren*ko D, Ono S, Lynch TJE, Swanson SD. Technical note: hydrogel-based mimics of prostate cancer with matched relaxation, diffusion and kurtosis for validating multi-parametric MRI. Med Phys. 2024;51(5):3590-6.

Supplementary Tables

Supplementary Table S1: Measured internal standard ADC values used to derive phantom scan temperature, Ts, using NIST calibration [https://data.nist.gov/od/id/mds2-2366]

ADC [SD] (μm2/ms)	Sys1-pt1	Sys1-pt2	Sys2-pt3	Sys2-pt4
**PVP20**	1.22 [0.02]	1.21 [0.02]	1.23 [0.02]	1.29 [0.01]
**water**	2.12 [0.05]	2.10 [0.06]	2.05 [0.04]	2.23 [0.04]
**Ts±0.5°C**	21.4	21.1	21.0	23.5

Average water diffusion coefficient, Dw, was determined using PVP-ADC calibration [2], Dw=ADCpvp/(K1·Cpvp2+K2·Cpvp+1), K1 = 12.5·1e-5; K2 = -23.2·1e-3, Cpvp=[0, 20]; and apparent scan temperature, Ta, was derived by inverting the Speedy-Angell relation [3]: Ta=T0·((Dw/D0)1/G+1)-273.15; T0=215.05K; G=2.063; D0=16.35mm^2/ms. Phantom Ts was derived as an average Ta for water and PVP20.

Supplementary Table S2: DK phantom reference values for repeated calibration (b = 0, 200, 500, 800, 1500, 2000, 2500 s/mm^2; Ts = 21.0 ± 0.5 °C).

Parameter mean [HW]	GS7	nTZ	nPZ	Atr
**ADC(DK) (μm2/ms)**	1.05 [0.05]	1.31 [0.06]	1.44 [0.03]	1.67 [0.04]
**ΔADCref [4]**	-0.01[0.01]	-0.02 [0.0]	0.02 [-0.01]	-0.05 [0.0]
**K**	0.98 [0.04]	0.87 [0.05]	0.75 [0.03]	0.48 [0.03]
**ΔKref [4]**	-0.07[-0.01]	-0.03 [0.0]	0.05[-0.02]	-0.02[-0.02]

Supplementary Table S3. ROI mean T_2 and ADC parameters for bp-phantom tissue mimics across different scan protocols

Protocol Mean parameter	GS7	nTZ	nPZ*	Atr
ADC ± 0.015 (μm^2/ms)				
**Sys1-pt1 (4b)**	0.64	0.91	1.03	1.33
**Sys1-pt2 (4b)**	0.77	0.92	1.07	1.31
**Sys2-pt3 (4b)**	0.79	0.88	1.03	1.36
**Sys2-pt4 (4b)**	0.83	0.92	1.07	1.43
**Sys1-pt1 (2b)**	0.52	1.09	1.18	1.39
**Sys1-pt2 (2b)**	0.92	1.10	1.22	1.41
**Sys2-pt3 (2b)**	0.97	1.07	1.24	1.48
**Sys2-pt4 (2b)**	1.02	1.10	1.27	1.58
**Mean [SD]**	0.81 [0.17]	1.0 [0.1]	1.13 [0.1]	1.41 [0.09]
**Mean(ADC-ADCref)**	-0.25	-0.33	-0.28	-0.31
**P**	0.005	0.2E-5	0.2E-5	0.8E-5
T_2 ± 1.5 (ms)				
**Sys1-pt1 (MEEPI)**	64	70	109	187
**Sys1-pt2 (MEEPI)**	73	79	100	166
**Sys2-pt3 (MESE)**	67	79	130	169
**Sys2-pt4 (MESE)**	73	82	139	178
**Sys2-pt3 (MEEPI)**	64	73	115	154
**Sys2-pt4 (MEEPI)**	67	76	121	160
**Mean [SD]**	68 [4]	77 [4]	119 [14]	169 [12]
**Mean(T_2-T_2ref)**	-3	-13	-18	-4
**P**	0.014	0.6E-3	0.014	0.62

GS7: Gleason7, TZ: transition-zone, PZ: peripheral-zone, Atr: atrophy tissue mimics; Sys-pt: system – patient protocol; SD: standard déviation ; ADCref : reference ADC value ; T_2ref : référence T_2 value ; P-value for one-way ANOVA comparison to bias corrected values (Table S4).

*One-way ANOVA test between 2b and 4b-protocol ADC values for nPZ phantom reference combined with lesions (Table 3, Figure 5B) before bias correction had P=0.044.

Supplementary Table S4. ROI mean T_2 and ADC parameters for bp-phantom tissue mimics corrected using bias-fit for different scan protocols with respect to DK reference values

Protocol<br>Mean parameter	GS7	nTZ	nPZ*	Atr
**ADC ± 0.015 (μm²/ms)**				
**Sys1-pt1 (4b)**	1.14	1.29	1.44	1.68
**Sys1-pt2 (4b)**	1.01	1.28	1.40	1.70
**Sys2-pt3 (4b)**	1.16	1.25	1.40	1.73
**Sys2-pt4 (4b)**	1.20	1.29	1.44	1.80
**Sys1-pt1 (2b)**	1.11	1.29	1.41	1.60
**Sys1-pt2 (2b)**	0.71	1.28	1.37	1.58
**Sys2-pt3 (2b)**	1.16	1.26	1.43	1.67
**Sys2-pt4 (2b)**	1.21	1.29	1.46	1.77
**Mean [SD]**	1.09 [0.17]	1.28 [0.02]	1.42 [0.03]	1.69 [0.08]
**Mean(ADC-ADCref)**	0.03	-0.05	0.00	-0.03
**T_2 ± 1.5 (ms)**				
**Sys1-pt1 (MEEPI)**	71	82	139	172
**Sys1-pt2 (MEEPI)**	80	91	130	171
**Sys2-pt3 (MESE)**	72	86	135	169
**Sys2-pt4 (MESE)**	78	89	144	173
**Sys2-pt3 (MEEPI)**	72	88	135	169
**Sys2-pt4 (MEEPI)**	75	91	141	175
**Mean [SD]**	75 [4]	88 [3]	137 [5]	172 [2]
**Mean(T_2-T_2ref)**	3.7	-1.2	0.3	-1.5

GS7: Gleason7, TZ: transition-zone, PZ: peripheral-zone, Atr: atrophy tissue mimics; Sys-pt: system – patient protocol; SD : standard deviation ; ADCref : reference ADC value ; T_2ref : référence T_2 value

*One -way ANOVA test between 2b and 4b-protocol ADC values for nPZ phantom reference combined with lesions (Table 3, Figure 5B) after bias correction had P=0.9.
